# Gunshot Wound Patterns: A Narrative Review From a Forensic Medical Perspective

**DOI:** 10.7759/cureus.101263

**Published:** 2026-01-10

**Authors:** Shahad A Alzahrani, Haya M Aldossari, Manal M Alessi, Sarah S Almutairi, Hitaf A Alqadoum, Ritesh G Menezes

**Affiliations:** 1 College of Medicine, King Fahd Hospital of the University, Imam Abdulrahman Bin Faisal University, Dammam, SAU

**Keywords:** firearm injuries, forensic investigation, forensic pathology, gunshot injuries, wound ballistics

## Abstract

Gunshot wounds rank among the frequently observed injuries in forensic pathology, possessing considerable legal and investigative relevance. Their varied manifestations, influenced by factors such as the weapon type, ammunition, distance of discharge, and trajectory of the projectile, necessitate a thorough forensic evaluation to ascertain the cause as well as the manner of death. This narrative review provides an overview of the classification and interpretation of gunshot wounds. Ultimately, this review underscores the vital role of gunshot wound analysis in forensic investigations and its importance in upholding justice and ensuring validity within legal contexts.

## Introduction and background

Overview of gunshot wounds

Gunshot wounds are intricate, violent, and traumatic wounds that are often seen in forensic practice [1]. Worldwide, injuries caused by firearms, either homicidal, suicidal, or accidental, contribute significantly to the overall number of deaths related to trauma, with thousands affected each year [2]. The impact of a projectile fired from a firearm as a result of gunpowder ignition causes these gunshot wounds. The characteristics and severity of these wounds can vary significantly based on several variables, including the firearm type, the caliber of the ammunition, the distance from which the firearm was discharged, and the specific body part that was struck [1]. The damage caused by a projectile can affect tissues in the permanent cavity it creates along its direct path and the surrounding tissues impacted by the temporary cavity. This temporary cavity forms as a result of short-lived but intense forces like radial acceleration, shear, stretching, and compression. While these forces act for only a short time, their impact on the tissues can be long-lasting [3]. Gunshot wounds are generally categorized into entry wounds, exit wounds, and occasionally re-entry wounds. Each type exhibits distinct forensic features, such as the abrasion collar, stippling, and soot deposition, that are vital for the analysis of wounds [1,3].

Importance of forensic analysis in gunshot wound cases

The forensic analysis of gunshot wounds plays a crucial role in medicolegal investigations. It allows forensic pathologists to ascertain essential details such as the distance and angle of the shot, the type of firearm used, and the probable manner of death, whether it was a homicide, suicide, or accident [3,4]. The characteristics of gunshot wounds often assist in reconstructing shooting events and can either corroborate or contradict eyewitness accounts in criminal proceedings [5]. Studies have highlighted the incidence of firearm-related injuries, with handguns being the most commonly employed weapon [4,5]. The head, chest, and limbs are frequently the affected regions [4,5].

Objective of the review

This narrative review intends to offer an overview of the forensic dimensions of gunshot wounds. It examines the patterns of gunshot wounds by analyzing how factors like firearm type and distance of firing affect the presentation of wounds. Furthermore, the review discusses the difficulties faced in interpreting wounds, including the impact of postmortem alterations and the constraints imposed by existing forensic technologies. Lastly, it evaluates the legal ramifications of gunshot wound analysis, specifically its importance in crime scene reconstruction and its relevance in judicial proceedings. A notable limitation of this narrative review is the lack of a comprehensive coverage of atypical gunshot wounds [6-8].

## Review

Patterns of gunshot wounds

Entry and Exit Wounds (Morphology and Influencing Factors)

Gunshot wounds are one of the commonly examined injuries in forensic pathology, which need thorough analysis to determine their characteristics and forensic relevance [3]. Distinguishing between entry and exit wounds is considered to be one of the most important aspects of the forensic examination of gunshot wounds, since their morphological features yield important information regarding the shooting event [9]. The study of such wound patterns enables the reconstruction of the circumstances under which the wound was sustained, determines both the range and distance of the firearm discharge, and identifies interactions with other objects [3].

Gunshot wounds are classified as entry and exit wounds, depending on the projectile's direction of travel. Entry wounds are typically smaller, with well-defined margins and tissue invagination. Edges of such wounds are usually inverted as a result of external trauma to the elastic dermal layer [3]. A unique ecchymotic ring with a purplish hemorrhagic rim around the injury might be observed as a result of blood vessel rupture in the dermis. However, this is not exclusive to entry wounds and may at times be observed in exit wounds as well [10]. Another important feature to mention is the abrasion collar surrounding the entry opening. This may also occur in exit wounds when the projectile encounters resistance during its exit [3]. However, exit wounds are generally characterized by outward bevelling of soft tissue with bleeding and rough, irregular margins. Other than that, these wounds usually have larger diameters compared to entry wounds. They also do not exhibit bullet wipe or secondary features that are usually seen in entry wounds, such as flame burns, singeing, tattooing, or a grease collar. However, in some cases, the ecchymotic ring and abrasion collar may still be found, particularly if the projectile meets resistance upon exit [3]. In addition, foreign material carried by the projectile, for instance, clothing fibres, bone fragments, or organ tissue, is generally present in the exit wound [11]. The presence of foreign material in exit wounds highlights the impact of the intervening structures. Histopathological examination usually shows a higher amount of fat infiltration compared to entry wounds. The effects of gunshot wounds are influenced by a variety of factors, such as the type of firearm, ammunition, and range of fire, interactions with other objects, and the projectile's behavior within the body. An understanding of these characteristics is important in forensic investigations since they provide valuable insights regarding the direction and range of fire, as well as possible intervening objects, hence helping in the reconstruction of the shooting events [3,12].

Powder Residue and Stippling Patterns

Gunshot residue, an important forensic marker used to reconstruct shooting incidents and ascertain firing distance, is composed of primer components (such as barium, antimony, lead, or other elements), burnt and unburned powder particles, and particles from the cartridge case, projectile, and firearm [13]. Gunshot residue can be detected in two ways: the first one is inorganically using flameless atomic absorption spectrometry, and the second is by particle analysis utilizing scanning electron microscopy with energy-dispersive X-ray (SEM-EDX) [13]. It is a standard procedure in forensic laboratory settings worldwide to prove the existence of such particles on the suspect's skin or clothing to further a criminal investigation or to provide evidence in a criminal prosecution [14].

Stippling, or tattooing, refers to a pattern of several tiny, punctuated abrasions in the skin around the entry wound [15]. Unburned gunpowder particles contacting the skin cause stippling, which occurs in close- to intermediate-range wounds [3]. Stippling cannot be washed off, in contrast to other substances that could accumulate on the skin, including soot. Soot weighs less compared to unburned gunpowder particles; as a result, most of it will only travel a few inches from the gun's muzzle before falling away. When stippling is present, it indicates that the gun's muzzle was within two feet of the victim's body at the time of fire [15].

Range of Firing and Wound Characteristics (Contact, Close-Range, Intermediate-Range, Distant)

The range of fire estimation is one of the important external factors considered in the examination of gunshot wounds. It helps forensic investigators to estimate the distance of the gun muzzle from the clothing or body of the victim when the gun was fired [15]. Estimation is mainly carried out by identifying the presence of gunshot residues, i.e., soot and unburned powder grains on clothing or skin. These substances are ejected from the muzzle together with the projectile and are deposited in patterns that relate to the range of fire [15].

Contact gunshot wounds occur when the muzzle of the gun is pressed against the skin during firing. They are commonly observed in suicidal firearm injuries [15]. This type of gunshot wound is associated with the deposition of soot and combustion gases deep within the wound, causing blackening of the initial wound track [16]. These wounds typically have a dense soot deposition pattern, which is not only within the wound but also around its borders when the contact is not firm. Soot can be removed by washing; therefore, forensic investigators must take photographs of it before washing the body for autopsy [15]. In firm (hard) contact wounds, nearly all the products of combustion pass through the wound, but in loose or angled contact, some soot is lost around the edges of the wound. The contact wound abrasion margin may appear seared or charred as a result of the heat and gases expelled from the weapon [15]. A muzzle imprint (a patterned abrasion or bruise), a key finding in contact wounds, can be seen, especially if the wound is on a bony part [16].

Close-range wounds are characterized by soot deposition and stippling. Stippling is a pattern of small abrasions and contusions surrounding the main entry wound caused by unburned gunpowder particles striking the skin. A firearm discharge within 2 feet (60 cm) of the victim's body can result in stippling, while soot is commonly observed at closer distances [16]. Stippling cannot be washed away, unlike soot, which can be removed. The stippling density and pattern may be used to estimate the distance of firing, and objects like clothing may alter the distribution [15,16].

Intermediate-range wounds occur when the firearm discharge is at a greater distance than close-range but still close enough that gunpowder particles reach the skin, but soot does not [16]. Stippling or tattooing of the skin is produced in this type of wound as a result of powder grains embedding and causing tiny abrasive and hemorrhagic lesions. The extent of tattooing depends on the distance, type of gunpowder/propellant (flake or ball powder, grain size), and surface characteristics of the skin or clothing. Some authors differentiate between "stippling" as impact marks and "tattooing" as forceful penetrations of powder grains. In cases where an interfering object (e.g., glass, fabric) filters out the gunpowder, pseudo-tattooing can occur, leaving irregular stippling patterns [16].

Distant-range wounds happen when the muzzle is so far away that neither soot nor powder grains reach the body. Such wounds are usually clean (externally) without surrounding residues. Nevertheless, the lack of soot or stippling by itself does not confirm a distant-range shot, since clothing or interfering objects can prevent residue deposition even in close-range shootings [15]. The minimum range of such gunshot wounds varies depending on the weapon, the ammunition, and the sensitivity of the investigation method used [16].

The most accurate method of determining the firing range is test-firing the same weapon using the same ammunition to compare the pattern of soot/stippling with autopsy findings. Moreover, clothing can also be analyzed for gunpowder residue to determine the maximum possible firing distance [15].

Wound Patterns by Firearm Type (Rifles, Shotguns)

Rifled firearms and smooth-bore firearms (shotguns) are the two types of firearms. Spiral grooves in the barrel of rifles impart spin to a single projectile (bullet), giving it stability and greater velocity. Smooth-bore firearms discharge several pellets within a cartridge, which scatter as they move, causing injuries that are more widespread but less penetrative with distance [3]. Because of these variations, different injury patterns are produced: rifled firearm, single bullet entry wound; smooth-bore firearm, multiple wounds caused by multiple pellets (distant-range) [3,17].

Depending on the weapon's type and firing range, different types of wounds are produced. Larger guns can emit flames up to 30 cm, whereas small arms can only emit flames up to 15 cm [3]. The firing distance affects the amount of smoke, soot, and stippling. Contact wounds may be loose or firm. Gases, gunpowder, flame, and other discharge elements primarily enter the body through contact. However, a tiny amount could be spotted on the skin close to the injury because of the loose contact. In firm contact, the wound is dependent on the underlying tissue. For instance, bone injuries cause stellate wounds because of gas pressure, whereas soft tissue wounds display burns and soot within but not on the skin [3].

The location of the wound has an impact on its look and severity. Damage to solid tissue and major organs is more serious. In addition, the projectile's velocity and residue deposition can be impacted by clothing and other objects [3]. Unless they are over the bone, rifled wounds are often circular, especially those due to contact shots. Entry wounds may exhibit an abrasion collar, contusion collar, and muzzle imprint. At close range, up to around 20 cm, burns and singeing of the hair caused by the flame, soot deposition, and tattooing are common [3]. Usually, exit wounds are bigger, more asymmetrical, and everted, especially when bones are broken [17]. A bullet (rifled firearm projectile) with high velocity creates cavitation, resulting in significant tissue damage [17]. On the other hand, a bullet fired at low velocity inflicts linear damage.

Shotguns fire pellets in a conical configuration [17]. Shotguns, like small arms, release smoke, flame, and unburned particles that affect the patterns of wounds [3]. Shotgun contact wounds have double muzzle imprints from the double barrels, when present [3]. Close-range wounds have clean edges, smoke soiling, circular abrasion rims, and circular entrance marks that are frequently the size of the muzzle. The wound may contain wadding or plastic containers [17]. Less tattooing is seen in intermediate-range wounds [17]. Tattooing disappears beyond 1 m. At distances of 2-3 m, a few pellet holes around a central wound are observed. Pellet distribution dominates the wound pattern as the distance increases. At greater distances (20-50 m), the pellet distribution creates superficial wounds that are rarely lethal [17]. Different weapons and ammunition have different pellet spreads, and barrel choking can have an additional impact on dispersion. Therefore, specific testing of the weapon and ammunition is necessary for range estimation [3].

Multiple Gunshot Wounds and Their Interpretation (Homicidal, Suicidal)

In forensic investigations, interpreting multiple gunshot wounds can be challenging [18]. Multiple gunshot wounds are traditionally seen as a reliable predictor of homicidal behavior, particularly when the wounds are spread over different anatomical locations. This inference is justified by the idea that a single self-inflicted wound is usually enough to result in death; therefore, the presence of several wounds is strongly suggestive of a second party's involvement [18]. Nevertheless, there are certain exceptions to this understanding.

Suicide by multiple gunshot wounds is rare, although it is conceivable, as several well-documented cases have shown. Suicidal persons have been known to cause multiple potentially lethal wounds to themselves using many firearms or in quick succession. It is worth noting that multiple gunshots to the head are uncommon in suicides, although multiple gunshot wounds to the chest are more common. Such patterns are frequently explained by a variety of circumstances, such as inadequate anatomical knowledge, involuntary flinching during firing, the use of faulty or incorrect ammunition, or the initial shot's inability to strike a key organ. In rare cases, people use two weapons at the same time to cause fatal head injuries [19].

When multiple gunshot wounds are present, several crucial characteristics from a forensic standpoint lend credence to the diagnosis of suicide. These include whether the firearm was found at the scene, whether the deceased's fingerprints or DNA were found on the weapon, and whether the wound sites were accessible: they typically need to be within the reach of the deceased's arm, which explains the fact that most suicidal gunshot wounds are contact wounds [17,19]. Additionally, the wounds are frequently found in areas of the body that are frequently linked to suicidal gunshot wounds, like the mouth, chin, chest, or temples, especially on the dominant side in the case of handguns [17]. Individuals hardly ever gunshot themselves in the abdomen, eyes, or inaccessible areas like the back.

The anatomical patterning commonly observed in suicides is absent from homicidal gunshot wounds. The distribution of wounds can vary greatly, and there is no preference for particular points of entry [18]. A homicidal manner of death is highly supported by the number and distribution of wounds, particularly when paired with other suspicious circumstances or the lack of a weapon at the scene [18].

Forensic challenges and considerations

Postmortem Changes Affecting Wound Analysis and Bullet Examination

Postmortem changes can cover injuries inflicted before death, making the interpretation of the cause and manner of death difficult. Decomposition, with its two types, autolysis and the other (putrefaction) caused by bacteria and fungi, causes skin changes such as skin color change, swelling, and blisters. So, premortem skin findings like contusions can be hard to recognize [20]. When a person gets shot, the bullet may stay within the body without exiting. If the bullet is not removed for a few days, it will be exposed to the chemicals inside the body caused by tissue breakdown. Our bodies have different chemical environments according to location or organ. For example, the stomach is considered to have a very high acidity concentration [21]. Hence, bullets could get damaged to different degrees according to the location and its conditions. An experimental study was conducted to study the effect of decomposition on bullets lodged in the body [21]. Two human cadavers, one placed in cool and the other in warm conditions, were used. Bullets were placed in both cadavers in three specific tissues, namely, the lower limb muscle, lung, and abdomen. These tissues were chosen due to different tissue configurations and chemical environments. The cadavers were left in the corresponding environment for 21 days to decompose [21]. From each tissue, three bullets were taken starting from the third day and every three days thereafter. The bullets were then examined by trained firearms examiners. The examination results indicated that the bullets from the cool environment cadaver were less affected by the slow decomposition process secondary to cool conditions [21]. On the other hand, the bullets extracted from the warm environment cadaver were harder to be examined microscopically due to fast decomposition secondary to warm conditions, as the insect and organic acids activity were higher in warm environments. Surface corrosion of bullets occurred faster when exposed to decomposition in warm conditions when compared to cool conditions [21].

Crime Scene Analysis and Postmortem Imaging

The investigation of fatal gunshot wounds has become more knowledgeable and proficient in recent years. Numerous facts and information have become forthcoming, and new techniques and scientific tools have been applied to the challenges associated with them [3,22,23]. One forensic method for examining and recreating the circumstances leading up to a crime is crime scene reconstruction [24]. To ascertain what happened, when it happened, and how it transpired, this procedure entails collecting, classifying, and analyzing physical evidence discovered at the crime scene. Because investigators carefully inspect the scene to find important evidence that could otherwise go unnoticed, it is key to successfully apprehending and convicting offenders [24].

Scene analysis: Before inspecting the body, the surroundings are observed and recorded, and pictures are taken. Any pointless actions that could change the body or the scene are avoided. The weapon, prescription drugs, and suicide note are looked for at the scene. The blood spatter is examined for hints regarding the event's dynamics, such as impact, transfer, or passive stains [25].

Handling the scene correctly is essential to protect forensic evidence and ensure accurate conclusions. The postmortem investigation process should begin at the scene, with a thorough understanding of the circumstances surrounding the death. To preserve trace evidence such as blood splatter or gunshot residue, the body must be handled as little as possible. Paper bags, rather than plastic ones, should be placed over the hands to prevent condensation and maintain any evidence present. During transfer, the body should be placed in body bags or clean linens to avoid direct contact with dirty surfaces. Additionally, the body should not be stripped at the morgue before the medical examiner conducts an inspection, as clothing may contain damage patterns or traces of evidence that correlate with the injuries on the body [19].

Postmortem imaging: To identify and locate possible bullets or their fragments, postmortem imaging is typically done before the autopsy. For every gunshot wound death, at least an X-ray examination should be conducted since bullets must be gathered during autopsy [25]. Postmortem computed tomography (CT) provides a 3D view of the body and is crucial for identifying entry and exit wounds, tracking the trajectory, and providing accurate details regarding organ damage and, consequently, the cause of death [26]. Additionally, it permits software modification of the CT scans, including 3D reconstruction, virtual probe addition, and measurement taking [25]. Moreover, CT has proven useful for recording and analyzing gunshot wounds [22,23]. A clear visual representation of the results can be crucial in courtroom situations [27].

Manner of Death Determination

Suicide is supported by a suicide note/letter, a history of mental disorders, and the presence of a weapon at the scene; nevertheless, an offender can mimic suicide by placing a weapon close to the victim's body or in the victim's hand [25]. Morphological results themselves cannot provide an accurate diagnosis. For instance, in a homicide, the temporal zone may have been specifically selected by the offender as a suicide-typical localization [28]. Moreover, a comprehensive medicolegal evaluation of all the information, including the findings of the autopsy and the investigation, is necessary for the shooter's identification.

Documentation, Expert Testimony, and Courtroom Challenges

Documentation: Because of the possible legal ramifications, thorough documentation is essential in forensic examinations. In addition to determining the cause and manner of death, the forensic pathologist must collect evidence that could implicate or clear suspects, establish identity if unknown, and ascertain the period of injury. In cases involving gunshot wounds, documentation should cover features of the entry wound, wound channel, and exit wound. If bullet fragments are found inside the body, their location must be documented. To prevent creating fake markings, they shouldn't be removed using metal forceps or any metal instruments. The direction of fire should be detailed using three axes: left/right, front/back, and top/bottom. Additionally, attention should be given to the extremities; a small, linear abrasion or bruise in the interdigital groove that runs between the thumb and index finger, known as a felc mark, may indicate that the skin was pinched, separating the handgrip from the sliding barrel, suggesting the shot was self-inflicted [19].

Expert testimony and medicolegal reliability: Forensic pathologists are essentially expert witnesses. Testimony must be believable if the entire scene and case history are known before autopsy, much like a physician needs to know a patient's medical history. The autopsy procedure must adhere to scientific guidelines to prevent the inclusion of erroneous or deceptive evidence [19]. After examination, fingerprinting should be done to protect trace evidence. In homicides, palm prints should also be obtained. X-rays are essential in all gunshot deaths, particularly to find jackets or bullet fragments that might not have left the body [19]. Appropriate collection and documentation of medical evidence are key to reliability in legal proceedings.

Avoiding procedural errors: Inappropriate body handling can compromise the reliability of forensic results in court. If the scene is handled too much, forensic evidence may be lost or destroyed. Also, missing clothing analysis, misplacing bullet fragments, and forgetting to record important injuries are examples of mistakes that might be used in court to discredit findings [19].

## Conclusions

Gunshot wound analysis is an essential component of forensic practice that provides vital information about the type of firearm used, the range of gunfire, and the cause of death. Important patterns of entry and exit wounds, residue deposition, and injury characteristics are compiled in this narrative review. Accurate interpretation is crucial for both crime scene reconstruction and judicial actions. Advancements in imaging and forensic technologies continue to enhance diagnostic accuracy. Ongoing research and standardized practices are vital to improving forensic investigations and supporting the delivery of justice.

## References

[1] Stefanopoulos PK, Pinialidis DE, Hadjigeorgiou GF, Filippakis KN (2017). Wound ballistics 101: the mechanisms of soft tissue wounding by bullets. Eur J Trauma Emerg Surg.

[2] (2025). Global status report on violence prevention 2014. https://www.who.int/publications/i/item/9789241564793.

[3] Shrestha R, Kanchan T, Krishan K (StatPearls Publishing). Gunshot wounds forensic pathology. StatPearls [Internet].

[4] Al Madni O, Kharosha MA, Shotar AM (2008). Firearm fatalities in Dammam, Saudi Arabia. Med Sci Law.

[5] Ghaffar UB (2024). Scenario of firearm injuries in Saudi Arabia: a comparative review. Cureus.

[6] Atreya A, Pokharel B, Khadka D, Basnet P, Gurung S, Hirachan N, Menezes RG (2025). Atypical gunshot injuries: a review of case reports and case series. J Forensic Leg Med.

[7] Linert B, Regnier J, Doyle BW, Prahlow JA (2010). Suicidal shotgun wound employing a shotgun barrel, a shotgun shell, and a BB. J Forensic Sci.

[8] Cunliffe CH, Denton JS (2008). An atypical gunshot wound from a home-made zip gun-the value of a thorough scene investigation. J Forensic Sci.

[9] Kislov MA, Chauhan M, Leonov SV, Pigolkin YI (2022). Forensic medical characteristics of firearm exit wounds in cases with armour protection. Leg Med (Tokyo).

[10] DiMaio VJ, DiMaio D (2001). Forensic Pathology. Forensic Pathology.

[11] Carr D, Kieser J, Mabbott A, Mott C, Champion S, Girvan E (2014). Damage to apparel layers and underlying tissue due to hand-gun bullets. Int J Legal Med.

[12] Baptista MV, d'Ávila SC, d'Ávila AM (2014). Histopathological detection of entry and exit holes in human skin wounds caused by firearms. J Forensic Leg Med.

[13] Shkrum MJ, Ramsay DA (2007). Forensic Pathology of Trauma: Common Problems for the Pathologist. Human Press Inc.

[14] Hannigan TJ, McDermott SD, Greaney CM, O'Shaughnessy J, O'Brien CM (2015). Evaluation of gunshot residue (GSR) evidence: surveys of prevalence of GSR on clothing and frequency of residue types. Forensic Sci Int.

[15] Denton JS, Segovia A, Filkins JA (2006). Practical pathology of gunshot wounds. Arch Pathol Lab Med.

[16] Houck MM (2017). Forensic Anthropology. Forensic anthropology.

[17] Payne-James J, Jones R, Karch SB, Manlove J (2011). Simpson's Forensic Medicine. Simpson’s Forensic Medicine. 13th ed. Hodder & Stoughton Ltd.

[18] Desinan L, Mazzolo GM (2005). Gunshot fatalities: suicide, homicide or accident? A series of 48 cases. Forensic Sci Int.

[19] Di Maio VJ (1999). Gunshot Wounds: Practical Aspects of Firearms, Ballistics, and Forensic Techniques. https://ia801209.us.archive.org/35/items/Gunshot_Wounds_Practical_Aspects_Of_Firearms_Ballistics_Forensic_Techniques/Gunshot_Wounds_Practical_Aspects_Of_Firearms_Ballistics__Forensic_Techniques.pdf.

[20] Vignali G, Franceschetti L, Attisano GC, Cattaneo C (2023). Assessing wound vitality in decomposed bodies: a review of the literature. Int J Legal Med.

[21] Bolton M, Chadwick S, Ueland M (2024). The effect of human decomposition on bullet examination. Forensic Sci Int.

[22] Zhang M (2022). Forensic imaging: a powerful tool in modern forensic investigation. Forensic Sci Res.

[23] Grabherr S, Egger C, Vilarino R, Campana L, Jotterand M, Dedouit F (2017). Modern post-mortem imaging: an update on recent developments. Forensic Sci Res.

[24] Lurigio AJ, Lenik J (2024). Crime Scene Reconstruction and Staging. EBSCO.

[25] Gitto L, Stoppacher R (2025). Gunshot wounds. https://www.pathologyoutlines.com/topic/forensicsgunshotwounds.html.

[26] Cascini F, Polacco M, Cittadini F, Paliani GB, Oliva A, Rossi R (2020). Post-mortem computed tomography for forensic applications: a systematic review of gunshot deaths. Med Sci Law.

[27] Oura P, Brix M, Lammentausta E (2024). Three-dimensional visualization of gunshot cavities in ballistic gelatine with computed tomography - a forensic ballistics case study. J Forensic Leg Med.

[28] Padosch SA, Schmidt PH, Schyma C, Hirsch RD, Kröner LU, Dettmeyer RB, Madea B (2004). Medicolegal aspects of witnessed suicide due to gunshot to the head. II. Legal medicine aspects and examination of the firing hand [Article in German]. Arch Kriminol.

